# Norcantharidin Inhibits cell growth by suppressing the expression and phosphorylation of both EGFR and c-Met in human colon cancer cells

**DOI:** 10.1186/s12885-016-3039-x

**Published:** 2017-01-13

**Authors:** Peiju Qiu, Siwen Wang, Ming Liu, He Ma, Xuan Zeng, Meng Zhang, Lingling Xu, Yidi Cui, Huixin Xu, Yang Tang, Yanli He, Lijuan Zhang

**Affiliations:** 1 Key Laboratory of Marine Drugs, Chinese Ministry of Education, School of Medicine and Pharmacy, Ocean University of China, 5 Yushan Road, Qingdao, 266003 China; 2 Institute of Cerebrovascular Diseases, Affiliated Hospital of Qingdao University, Qingdao, 266003 China

**Keywords:** Norcantharidin, Colon cancer, Cell cycle, Apoptosis, EGFR, c-Met

## Abstract

**Background:**

Norcantharidin (NCTD) is a Chinese FDA approved, chemically synthesized drug for cancer treatment. The effect of NCTD on signaling proteins of EGFR and c-Met was systematically elucidated in current study.

**Methods:**

Two human colon cancer cell lines, HCT116 and HT29, were used as model systems to investigate the anti-cancer molecular mechanism of NCTD. Cell cycle arrest and early/late apoptosis were analyzed by flow cytometry. The levels of EGFR, phospho-EGFR, c-Met, phospho-c-Met and other related proteins were quantified by western blot analysis.

**Results:**

NCTD induced cell cycle arrest at G2/M phase in both cell lines. The early and late apoptosis was also observed. Further investigation indicated that NCTD suppressed not only the expression of the total EGFR and the phosphorylated EGFR but also the expression of the total c-Met and the phosphorylated c-Met in colon cancer cells. Moreover, EGFR expression could be mostly restored by co-treatment with MG132, a proteasome inhibitor. In addition, NCTD-induced cell death was comparable to that of the anti-cancer drug gefitinib, a tyrosine kinase inhibitor for EGFR, based on the immunoblot analysis of the expressed proteins after the drug treatment.

**Conclusions:**

NCTD might be a useful and inexpensive drug candidate to substitute for gefitinib to serve the treatment needs of cancer patients.

## Background

**Fig. 1:**
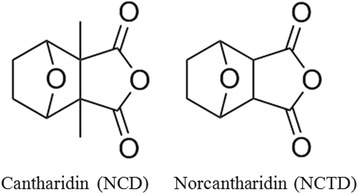
The structure of norcantharidin (NCTD) and cantharidin (CTD)

Cantharidin (CTD) is isolated from Mylabris phalerata Pall, a valued traditional Chinese medicine in treating skin problems, such as furuncles and piles, for more than 2000 years in China. In recent years, CTD has been identified as the active compound in Mylabris phalerata Pall with anti-tumor activity [1–4]. However, it causes severe side effects in the urinary system both in animals and in human [5]. Norcantharidin (NCTD), which is chemically demethylated from CTD (Fig. 1), overcomes the urological toxicity while enhancing its anti-tumor activity [6]. Based on the cinical studies, NCTD has been approved by Chinese FDA for liver, esophageal, and gastric cancer treatment in China since 1996.

Numerous studies have indicated that NCTD possessed the abilities to induce cell death for multiple types of cancer cells, for example, colorectal and lung cancer cells. The anti-cancer activity of NCTD relies on its ability in activation of the caspase signaling pathway that leads to apoptosis [7] accompanied with a decreased ratio of Bcl-2/Bax [8, 9], the changes in the expression of cell cycle-related proteins are co-related with cell cycle arrest [10], the interruption of DNA synthesis [11], inhibition of tumor invasion and metastasis [12, 13], MAPK activation, and protein kinase C pathway activation [14].

Colon cancer is the fourth most common cancer in the world found in both men and women. It is well known that two cell surface receptor tyrosine kinases, c-Met and EGFR, are co-present in 78% [15] to 80% [16, 17] in colon cancer cells. EGFR and c-Met corroborate the downstream signaling pathways in cancer cells in that c-Met and EGFR activate many of the same downstream signaling molecules. c-Met signaling is mainly responsible for cancer cell survival when EGFR inhibitors, such as gefitinib, are used during cancer treatment [18]. So far, few c-Met inhibitors or c-Met pathway antagonists have been developed into drugs. Even though several novel compounds are in clinical trials, their potential use might be limited by their potency, pharmacokinetic defect, and safety profile [19]. Therefore, it is highly desirable to find dual inhibitor for both EGFR and c-Met, especially from the existing drugs, to facilitate colon cancer treatment.

In current study, we demonstrated that NCTD suppressed the expressing level and phosphorylation of both c-Met and EGFR. Moreover, NCTD-induced cell death was comparable to that of the anti-cancer drug gefitinib, a tyrosine kinase inhibitor for EGFR, based on the immunoblot analysis of the expressed proteins after the treatment of NCTD.

## Methods

### Cell-related experiment and NCTD treatment

HT29 and HCT116, two human colon cancer cell lines, were obtained, maintained, and passaged using the identical protocol described in the published report [20]. NCTD was obtained from National Institutes for Food and Drug Control (Shandong, China). NCTD was dissolved in dimethylsulfoxide (DMSO) and the final concentration of DMSO used was 0.1% in culture media for all experiments.

### Cell growth inhibition assay

HT29 and HCT116 cells suspended in complete media were seeded in 96-well plates (2000 cells/well). After growing for 24 h in incubator, media were aspirated and 0.2 mL complete media containing serial concentrations of NCTD were added to each well. After incubating the plate for 24, 48, or 72 h, 20 μL of resazurin (2 mg/mL dissolved in water, Sigma) was added to each well. The fluorescent signal was monitored using 544 nm excitation and 595 nm emission wavelengths by Spectramax M5 plate reader (Molecular Devices) after incubation at 37 °C for 16 h in the incubator. The number of living cells in each well was proportional to the relative fluorescence unit (RFU) measured by the assay [20].

### Cell cycle analyses

HCT116 and HT29 cells were added in 6-well plates with 6 × 10^4^ cells/well with 2 mL complte media per well. After incubation for 24 h, different concentrations of NCTD in 2 mL of complete media was added to treat cells. After 24 h incubation, the cell pellets were collected using the published protocol [20] and resuspended in 1 mL of 70% ethanol at 4 °C overnight. For analysis, the supernatant was removed by centrifuging at 1600 g for 1 min. The cell pellets were incubated with 0.5 mL PI/RNase Staining Buffer (BD Biosciences) for 15 min at room temperature. Single-cell suspension was generated by gentle pipetting. The cells was analyzed using Beckman cell analyzer FC500-mpl. Beckman CXP software and Multicycle software were used for data analysis.

### Apoptosis assay

Annexin V/propidium iodide (PI) double staining assay (BD Biosciences, San Diego CA, USA) was used to quantify the apoptotic cells by following the manufacturer’s instruction [21]. Briefly, HCT116 and HT29 cells were treated with different concentration of NCTD in complete cell culture media for 24 h. After removing NCTD-containing media, cells were incubated in 100 μL buffer containing Annexin V and PI at room temperature for 15 min. Beckman cell analyzer FC500-mpl was then used to analyze the apoptotic cells.

### Western blot analysis

HCT116 and HT29 cells were cultured in 10-cm dishes for 24 h before treated with different concentrations of NCTD or NCTD plus MG132 (Beyotime biotechnology, China). After being incubated with another 72 h, the cells were collected with cell-scrapers into 1.5 mL tube and put on ice for 30 min in whole cell lysis buffer (Cell Signaling Technology, USA) containing protease inhibitors. Proteins were quantified by BCA protein assay kit. Equal amount of proteins from different sample (50 μg) were resolved over 8 or 12% SDS-polyacrylamide gel by electrophoresis and then transferred to nitrocellulose membrane. The membranes were put in blocking buffer at room temperature for 2 h before appropriate primary antibody was added. The membrane was incubated in the presence of primary antibody at 4 °C overnight before the corresponding secondary antibodies were added. The membranes were then visualized by using Western Lightning. Antibodies for EGFR, Phospho-EGFR (Tyr1068), Her-2, Phospho-Her2/ErbB2 (Tyr1248), c-Met, Phospho-c-Met (Tyr1003), Caspase-3, Rb, β-Actin, PARP, CDK-2, CyclinD1, and Bax were purchased from Cell Signaling Technology (USA).

## Results

### NCTD inhibited HCT116 and HT29 cell proliferation

**Fig. 2:**
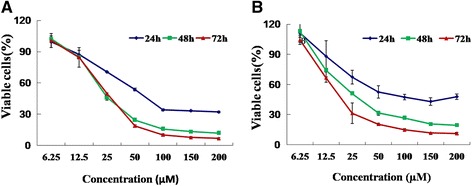
Effect of NCTD on cell viability. HCT116 (**a**) and HT29 (**b**) human colon cancer cells were seeded in 96-well plates with 2000 cells/well, respectively. After 24 h, cell culture media were replaced with 200 μL complete media containing serial concentrations of NCTD to treat the cells within the time of period indicated. The viable cells were measured by the fluorescent signal as described in the Material and Methods Section. The experiments were repeated for three times

The inhibitory effects of NCTD on cell proliferation of HCT116 (Fig. 2a) and HT29 (Fig. 2b) were detected by the resazurin assay. As shown in Fig. 2, NCTD induced time- and concentration-dependent cell death in both HCT116 and HT29 cells. Clinically, NCTD (Mw 168) comes either as tablets to take by mouth (5-15 mg, 3 times a day) or to take by injections (10–20 mg/day). A patient generally has 4 to 5 l of blood. Therefore, the maximum concentrations of NCTD in patient’s blood should be around 4–5 μg/mL (24–30 μM). The data in Fig. 2 showed that 100 μM (16.8 μg/mL) NCTD induced >50% cell death in both cancer cell lines at all the time (24, 48, and 72 h) points tested. Based on the data, NCTD were further studied from 6.25 to 100 μM in both cancer cell lines for different types of tests.

### NCTD suppressed the expression and activation of c-Met and EGFR in HCT116 and HT29 cells

**Fig. 3:**
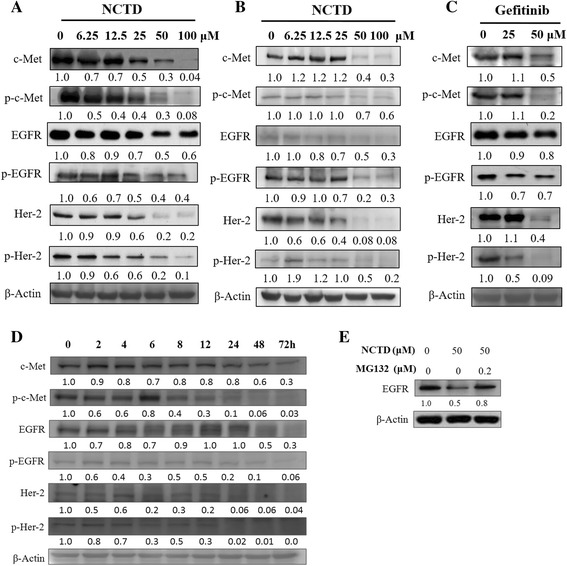
Effects of NCTD on the expression and activation of EGFR and c-Met on HCT116 (**a**) and HT29 (**b**) human colon cancer cells. The cells were seeded in 10-cm dishes for 24 h, and then cells were treated with NCTD or gefitinib (**c**) at 6.25, 12.5, 25, 50, and 100 μM, respectively. After 72 h of incubation, cells were harvested for western blot analysis. Cells were treated with 100 μM of NCTD for the indicated periods after which the cells were collected and prepared for western blot analysis (**d**). HCT116 cells were treated with 50 μM NCTD or 50 μM NCTD plus 0.2 μM MG132 for 48 h (**e**). The expression of EGFR or β-Actin protein was detected by western blot analysis. The numbers underneath the blots represent band intensities (normalized to the loading controls, means of three independent experiments) measured by the Image J software. The standard deviations were all within ±15% of the means (data not shown). β-Actin was used as equal loading controls. The experiments were repeated for three times

To understand the NCTD-induced cytotoxicity, we tested the activation and expression of the EGFRs and c-Met after NCTD treatment. As shown in Fig. 3a, NCTD at 25 μM suppressed the phosphorylation of EGFR (Tyr1068) by 50%, however, NCTD moderately decreased the level of EGFR even up to 100 μM. Her-2 is another predominant member of EGFR family. Treatment of NCTD also significantly decreased the level of Her-2 and phosphor-Her-2 in dose-dependent manner. Importantly, NCTD substantially decreased expression of both phospho-c-Met and total c-Met. For example, NCTD at 6.25, 25, 100 μM decreased the level of phosphor-c-Met by 50, 60, 92% while inhibited the expression of total c-Met by 30, 50, 96%, respectively. Parallelly, HT29 responded to NCTD in the same trend, i.e., NCTD suppressed the activation of EGFR, Her-2 and c-Met in dose-dependent manner (Fig. 3b).

To evaluate the potency of NCTD in killing colon cancer cells, we used gefinitib, the EGFR inhibitor, as the positive control. We compared the effect of gefinitib on attenuating the expression of the same group proteins, i.e., EGFR, Her-2 and c-Met, at two concentrations of 25 μM and 50 μM, respectively. Based on the protein band intensities, we found that NCTD exhibited stronger inhibitory effect on phosphorylation of c-Met, EGFR, and Her-2 at the concentration tested despite the expression of phosphor-Her-2 at 50 μM. Altogether, we concluded that NCTD was comparable to gefinitib in attenuating colon cancer cell growth (Fig. 3c).

In a time-dependent study, HCT116 cells were treated by NCTD at 100 μM for a period up to 72 h. The same group of proteins including c-Met, EGFR, Her-2, were analyzed again by western blot analysis. Decrease of total and phospho-c-Met, EGFR, Her-2 occurred in the 6 to 24 h and persisted for up to 72 h (Fig. 3d) by NCTD treatment.

The mechanisim of the downregulation of EGFR induced by NCTD in HCT116 cell was further invesitigated by co-treatment in combination with MG132, a proteasome inhibitor. As showed in Fig. 3e, NCTD induced 50% decrease in EGFR expression while NCTD plus MG132 induced 20% decrease in EGFR which indicated NCTD inhibited EGFR expression mainly via proteasome-mediated degradation.

### G2/M cell cycle arrest induced by NCTD

**Fig. 4:**
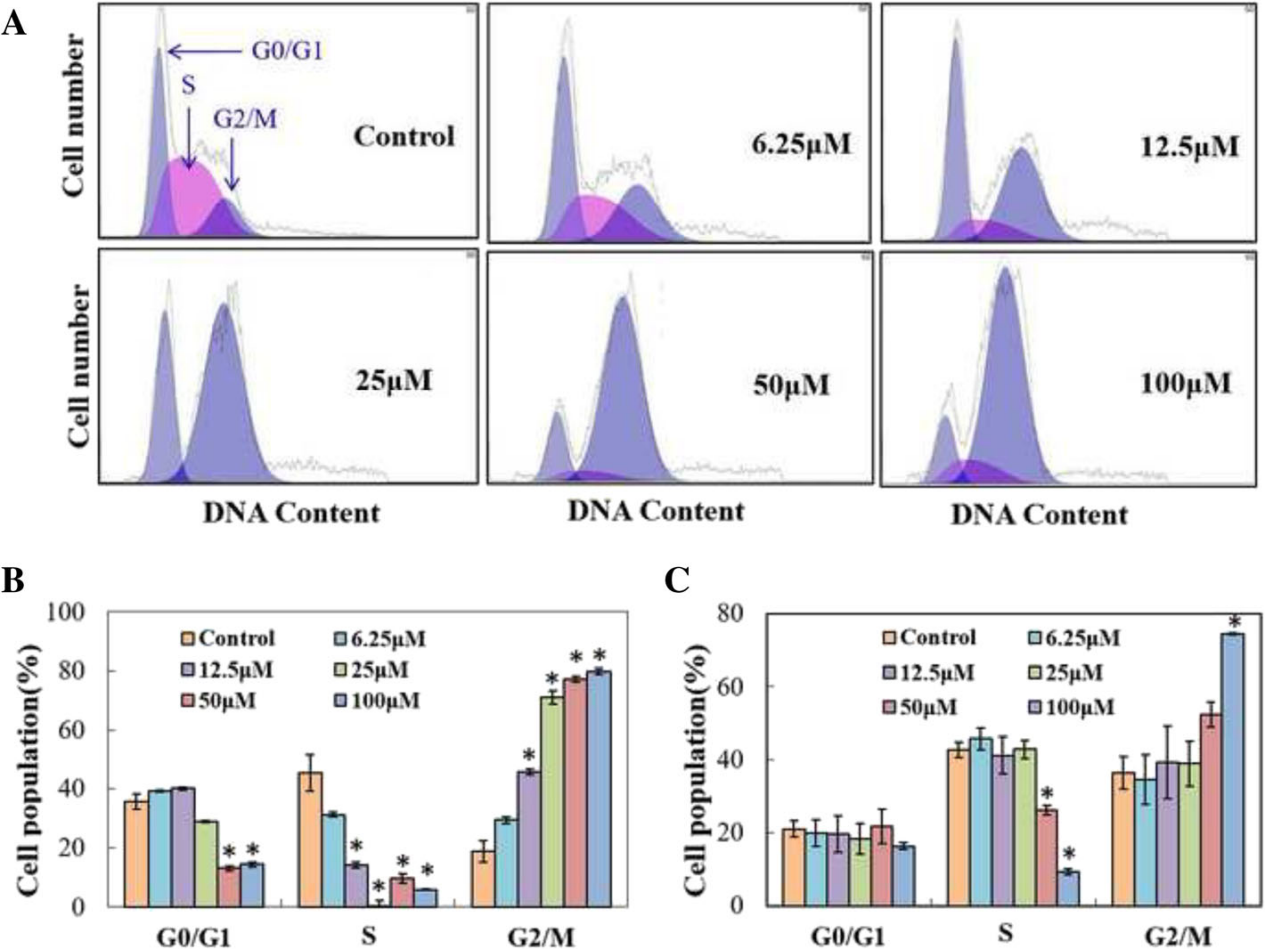
Cell cycle distribution of HCT116 (**a**) and HT29 (**b**) human colon cancer cells after treatment with NCTD. The cells were seeded in 6-well plates for 24 h and then treated with NCTD in complete media. After 24 h treatment, cells were harvested and subjected to cell cycle analyses as described in Materials and Methods. All data represent mean ± SD. Statistical analysis were conducted among control and treated groups in G0/G1, S and G2/M phases separately. Statistical significance was determined by Student’s *t* test (*p* < 0.001). * represent significant difference in cell number in the control that received PBS versus those treated with the indicated concentration of NCTD. Each bar represents the average value ± S.D; *n* = 3

To understand how NCTD induced cell death in both cell lines, flow cytometry assay was used to study the effect of NCTD on cell cycle after treating the cells with varied concentrations of NCTD. NCTD caused G2/M phase cell population accumulation in a dose-dependent manner in HCT116 cells compared to that of control (Fig. 4b), which was consistent with previous report [22]. For example, at the concentration of 12.5 and 50 μM, NCTD induced an increase in G2/M phase cell population by 2.2- to 4-fold, respectively. Similar results were observed in HT29 cell line when the concentration of NCTD was above 50 μM. Interestingly, the cell population of G2/M phase had no change when the NCTD concentrations were below 25 μM (Fig. 4c).

### NCTD induced early/late apoptosis

**Fig. 5:**
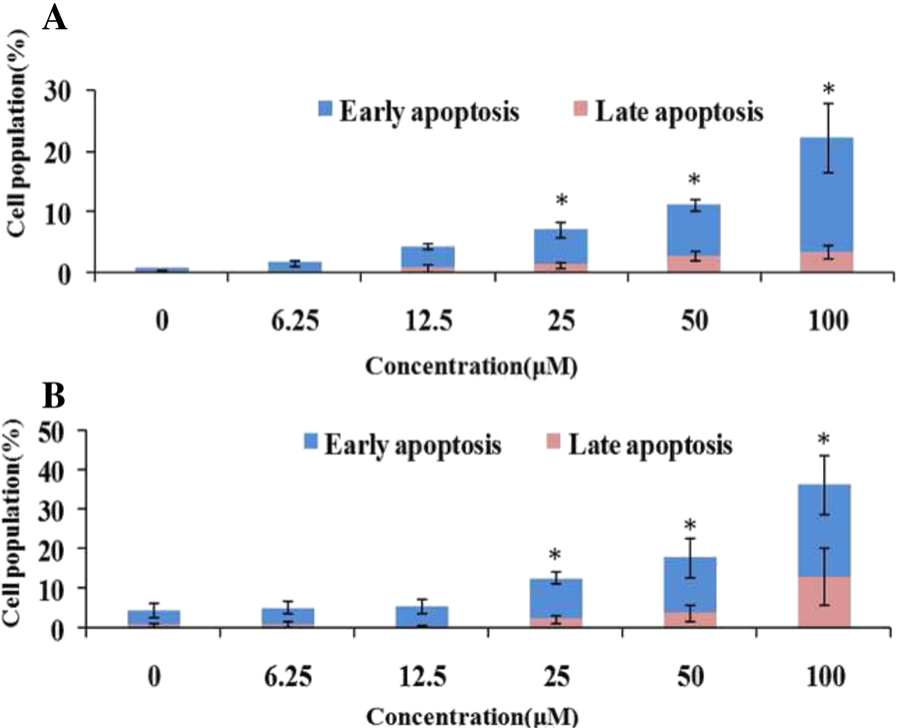
Effect of NCTD on apoptosis in HCT116 (**a**) and HT29 (**b**) cells. The cells were seeded in 6-well plates for 24 h, and then the cells were treated with serial concentrations of NCTD. After 48 h of treatment, cells were harvested and subject to apoptosis analyses as described in Materials and Methods. The experiments were repeated for three times. Statistical significance was determined by Student’s *t* test (*p* < 0.001). * represent significant difference in cell number in the control that received PBS versus those treated with the indicated concentration of NCTD. Each bar represents the average value ± S.D.; *n* = 3

We also measured the degree of apoptosis induced by NCTD. As shown in Fig. 5a, compared to the control, treating HCT116 cells with NCTD increased the percentage of early and late apoptotic cells significantly in a concentration-dependent manner (Fig. 5a). For example, NCTD at 100 μM increased early apoptotic cell population by 38.44-fold over the control cells, which was similar for HT29 cells (Fig. 5b).

### NCTD affected cell cycle- and apoptosis-related proteins

**Fig. 6:**
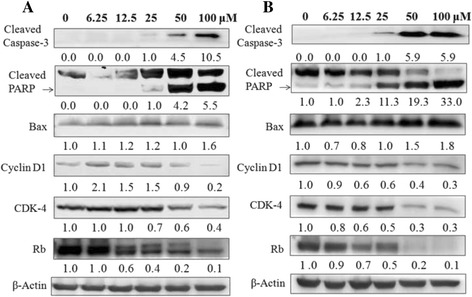
Effects of NCTD on the key proteins regulating cell cycle and apoptosis in HCT116 (**a**) and HT29 (**b**) human colon cancer cells. The cells were seeded in 10-cm dishes for 24 h and then treated with different concentrations of NCTD. After 72 h of incubation, cells were collected for western blot analysis as described in the Materials and Methods. The numbers underneath of the blots represent band intensity (normalized to β-Actin, means of three independent experiments) measured by Image J software. The standard deviations (all within ± 15% of the means) were not shown. β-Actin was served as an equal loading control. The experiments were repeated for three times

How NCTD affected cell cycle- and apoptosis-related signaling proteins was tested over a range of concentrations from 6.25 to 100 μM in both cell lines for 72 h. At 25 μM concentration of NCTD, the level of cleaved PARP were increased significantly and the cleaved caspase-3 was also to show up (Fig. 6a). However, another apoptosis-related protein, Bax, barely changed under this concentration. Meanwhile, the decrease on several cell cycle-related proteins including CyclinD1, Rb, CDK-4 was observed after treatment with 12.5 to 100 μM of NCTD. In addition, similar trends were detected when HT29 cell line was used to perform the same test. In conclusion, NTCD affected both cell cycle- and apoptosis-related signaling proteins in a concentration-dependent manner (Fig. 6b).

## Disscussion

Accumulating evidences indicated that both c-Met and EGFR were overexpressed by 78 to 80% of colon cancers, which were associated with poor outcome. A cross-talk of c-Met and EGFR could modulate reciprocally and eventually determine the intensity of c-Met signaling pathway [18]. One of the major findings of our study was that the mechanism underlying the cell death induced by NCTD involved in suppressing the expression and phosphorylation of c-Met and EGFR. To our knowledge, this is the first demonstration that NCTD was a dual inhibitor for c-Met and EGFR and in human colon cancers.

Another interesting finding was that signaling network might also exist between c-Met and Her-2 in colon cancer cells where the level of the two proteins decreased with the increased concentration of NCTD. However, we could not draw a conclusion how c-Met affected the expression and activation of Her-2, such as by direct suppression or indirect regulation. Additional studies were necessary to reveal the potential mechanism how c-Met downregulated Her-2 expression in colon cancer cells line.

**Fig. 7:**
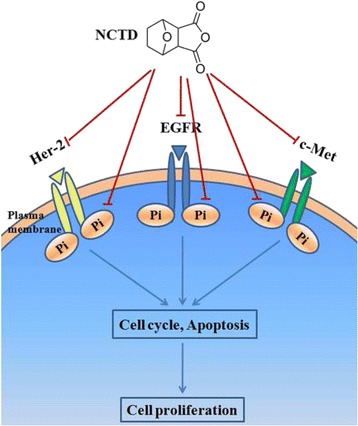
Proposed molecular mechanisms by which NCTD inhibited human colon cancer cell growth

With the dramatic effects against lung cancer, gefitinib has been the most used small molecular EGFR inhibitor. To evaluate the potency of NCTD in killing colon cancer cells, gefitinib was used as the positive control in our study. Our data showed that NCTD was better at suppressing the phosphorylation of EGFR tested at 25 μM while gefinitib exhibited stronger inhibitory effect on the phosphorylation of Her-2 at 50 μM. Collectively, the data suggested that NCTD might have a different mechanism from gefitinib in killing colon cancer cells. Remarkably, although the two drugs exhibited comparative effect on attenuating the EGFR, Her-2, and c-Met, NCTD seemed to possess certain advantages over gefitinib including lower cost, better safety, and superior tolerance of NCTD [6] (Fig. 7).

## Conclusions

In conclusion, NCTD suppressed the phosphorylation and expression of both EGFR and c-Met in HCT116 and HT29 human colon cancer cells. Our data provided novle molecular mechanism for further investigation if NCTD could serve as a dual inhibitor for EGFR/c-Met in terms of colon cancer treatment.

## Abbreviations

CTD: Cantharidin
NCTD: Norcantharidin
PI: Propidium iodide
RFU: Relative fluorescence unit
